# Physical activity relates to carotid plaque vulnerability in older persons with subclinical carotid atherosclerosis

**DOI:** 10.1016/j.ebiom.2025.105894

**Published:** 2025-08-20

**Authors:** Luoshiyuan Zuo, Maryam Kavousi, Julie A.E. Van Oortmerssen, Trudy Voortman, M Kamran Ikram, Daniel Bos

**Affiliations:** a Department of Epidemiology, Erasmus MC, University Medical Centre Rotterdam, Rotterdam, the Netherlands; b Meta-Research Innovation Centre at Stanford (METRICS), Stanford University, Stanford, USA; c Department of Neurology, Erasmus MC, University Medical Centre Rotterdam, Rotterdam, the Netherlands; d Department of Radiology and Nuclear Medicine, Erasmus MC, University Medical Centre Rotterdam, Rotterdam, the Netherlands

**Keywords:** Carotid atherosclerosis, Plaque composition, Physical activity, MRI, Stroke

## Abstract

**Background:**

Recent evidence suggests that excessive physical activity may accelerate the progression of coronary atherosclerosis. However, data on carotid atherosclerosis remains scarce. This study aimed to evaluate the association between physical activity and changes in carotid atherosclerotic plaque vulnerability, and the interaction between these two variables with the risk of first-ever stroke.

**Methods:**

This prospective study included 1330 stroke-free persons (mean age: 71.5 years) from the population-based Rotterdam Study with carotid atherosclerosis. Carotid magnetic resonance imaging (MRI) was performed to assess intraplaque haemorrhage (IPH) and lipid-rich necrotic core (LRNC), both recognised as important vulnerable plaque components, with a follow-up MRI conducted approximately six years later in 699 participants. Physical activity was assessed using a questionnaire at baseline MRI. The association of physical activity with incident plaque components and the interaction between these two variables with the risk of stroke were analysed, adjusting for socioeconomic status and conventional cardiovascular risk factors.

**Findings:**

Higher volumes of total, moderate-to-vigorous, and vigorous physical activity were associated with an increased risk of incident IPH and LRNC, with adjusted odds ratio (OR) ranging from 1.08 to 1.35 per 20 metabolic equivalent of task (MET)-hours/week increase. Physical activity was further categorised using literature-based cut-offs, tertiles, quartiles, and quintiles, with the lowest group as the reference. The risk of incident IPH was significantly higher exclusively in the top quintiles of total, moderate-to-vigorous, and vigorous physical activity (adjusted OR range: 1.87–2.54, all P < 0.05), with thresholds of potential harm (99, 70, and 26 MET-hours/week, respectively) substantially exceeding current guideline recommendations for cardiovascular disease prevention (15, 15, and 7.5 MET-hours/week, respectively); similar patterns were also observed for LRNC. No association was found for moderate activity. The association between physical activity and first-ever stroke differed by the presence of vulnerable plaque components at baseline (P for interaction = 0.010 for total, 0.095 for moderate-to-vigorous), and physical activity inversely associated with stroke only in individuals without vulnerable plaque components (adjusted hazard ratio range: 0.69–0.71, all P < 0.05).

**Interpretation:**

In older individuals with pre-existing carotid atherosclerosis, the most physically active group may have a higher risk of developing vulnerable carotid plaques. Individuals with pre-existing vulnerable carotid plaques may lose the benefits of physical activity.

**Funding:**

The Rotterdam Study is supported by the 10.13039/501100003061Erasmus Medical Center and 10.13039/501100001828Erasmus University Rotterdam; the 10.13039/501100003246Netherlands Organisation for Scientific Research (NWO); the 10.13039/501100001826Netherlands Organisation for Health Research and Development (ZonMw); the Research Institute for Diseases in the Elderly (RIDE); the 10.13039/501100024871Netherlands Genomics Initiative (NGI); the Ministry of Education, Culture and Science, the Ministry of Health, Welfare and Sports; the 10.13039/501100000780European Commission (DG XII); and the Municipality of Rotterdam. This work was further supported by the 10.13039/501100001674Leducq Foundation COMET Network. The funder of the study had no role in study design, data collection, data analysis, data interpretation, writing, or submission of the report.

**Research in context**

**Evidence before this study**

Prior studies suggested a curvilinear relationship between physical activity and cardiovascular disease. In particular, among persons with subclinical atherosclerosis or a history of cardiovascular disease, excessive physical activity may lose benefits and even cause harm. Notably, recent studies have indicated that athletes engaged in long-term intensive exercise may have a higher burden of coronary atherosclerosis. However, the impact of physical activity on changes in carotid atherosclerosis and the subsequent risk of stroke in community-dwelling individuals remains unclear.

**Added value of this study**

This prospective study included participants with subclinical carotid atherosclerosis from the population-based Rotterdam Study. The vulnerable carotid plaque components, including intraplaque haemorrhage and lipid-rich necrotic core, were measured using magnetic resonance imaging at baseline and six-year follow-up. We found that higher total, moderate-to-vigorous, and vigorous physical activity were associated with an increased risk of incident vulnerable components, particularly for those in the highest groups of physical activity. No association was observed for moderate physical activity. Furthermore, the association of physical activity with stroke differed by the presence of vulnerable carotid plaque; a favourable association was observed only in persons without vulnerable carotid plaques.

**Implications of all the available evidence**

Our findings suggest that excessive physical activity, particularly vigorous physical activity, may increase the vulnerability of pre-existing carotid plaques in older adults. Individuals with vulnerable carotid plaques may lose the benefits of physical activity and warrant special attention to its potential adverse effects. These results challenge the current “one-size-fits-all” approach of physical activity in the primary prevention of cardiovascular disease and highlight the need to define an upper limit of physical activity for persons with subclinical atherosclerosis.

## Introduction

Physical activity is an important—perhaps the most important—modifiable factor in the prevention of cardiovascular diseases (CVD).^1^ Current primary and secondary CVD prevention guidelines recommend >150 min of moderate-level and/or >75 min of vigorous-level physical activity every week without specifying an upper limit.^2^, ^3^, ^4^, ^5^ However, concerns have been raised about this “one-size-fits-all” approach as debates persist on the nonlinear relationship of physical activity with CVD.^6^, ^7^, ^8^, ^9^, ^10^, ^11^, ^12^ Particularly among persons already with atherosclerosis or CVD, some studies indicated that excessive physical activity may lose benefits and even cause harm.^13^, ^14^, ^15^

Within the pathophysiological framework of CVD,^16^^,^^17^ the rupture of vulnerable atherosclerotic plaque is firmly established as the primary cause. Physical activity, especially vigorous-level exercise, and the associated sympathetic neurohormonal activation can act as a trigger for plaque rupture, leading to sudden cardiac death.^16^^,^^18^^,^^19^ Notably, recent studies have suggested that athletes engaged in long-term intensive exercise may have a higher burden of coronary atherosclerosis compared to healthy non-athlete contrast.^20^, ^21^, ^22^, ^23^, ^24^ In this light, shifting the focus to carotid atherosclerosis could also be important for the primary prevention of CVD, as carotid atherosclerotic plaque affects over one-third of the general population aged 65 and over worldwide.^25^ With the presence of plaque at the carotid bifurcation, the acute haemodynamic responses to physical activity—such as elevated systolic blood pressure and turbulent blood flow—may transiently exert harmful effects, increasing plaque vulnerability.^26^^,^^27^ However, data regarding the influence of physical activity on changes in carotid plaque vulnerability and the subsequent risk of stroke in community-dwelling individuals remains scarce.

Studies using carotid MRI have demonstrated that plaque vulnerability is predominantly determined by its intrinsic components rather than its size or luminal stenosis.^28^, ^29^, ^30^, ^31^ In particular, intraplaque haemorrhage (IPH) and lipid-rich necrotic core (LRNC) are recognised as key features of high-risk plaques, strongly associated with first-ever stroke among individuals with no or low-degree carotid stenosis.^32^^,^^33^ Therefore, this study investigated the association between physical activity and changes in carotid plaque vulnerability, indicated by the incidence of IPH and LRNC in community-dwelling older adults with subclinical atherosclerosis. Additionally, we assessed whether the association between physical activity and the risk of stroke differed by the presence of vulnerable plaque components.

## Methods

### Study population

**Fig. 1:**
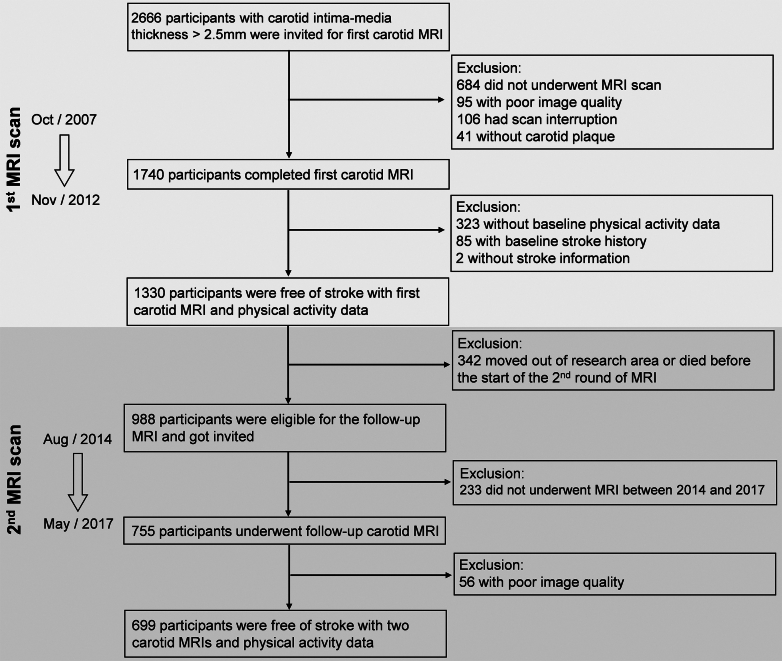
**Flowchart of participant selection**.

This prospective study was embedded within the Rotterdam Study, an ongoing prospective population-based cohort study which has been described previously.^34^ In brief, the Rotterdam Study was initiated in 1990, and extended recruitments of participants were performed in 2000, 2006, and 2016. By the end of 2008, the Rotterdam Study comprised 14,926 participants aged 45 and over who lived in the Ommoord district. Upon study enrolment and subsequent follow-up visits every 3–6 years, participants undergo extensive examinations at a dedicated research centre, including assessment of carotid intima-media thickness (IMT) via ultrasonography. Between 2007 and 2012, participants with carotid IMT >2.5 mm (n = 2666) were invited for carotid MRI to evaluate plaque components, resulting in eligible MRI images of 1740 participants. Following the exclusion of individuals with a history of stroke and those lacking physical activity data, the baseline population for the present study comprised 1330 participants. A follow-up carotid MRI was conducted approximately six years later. After excluding participants who relocated out of the research area or died before the initiation of follow-up MRI, 699 had eligible follow-up MRI images (Fig. 1).

### Ethics

The Rotterdam Study has been approved by the Medical Ethics Committee of Erasmus MC (registration number MEC 02.1015) and by the Dutch Ministry of Health, Welfare and Sport (Population Screening Act WBO, licence number 1071272-159521-PG). The Rotterdam Study Personal Registration Data collection is filed with the Erasmus MC Data Protection Officer under registration number EMC1712001. All participants provided written informed consent to participate in the study and to have their information obtained from treating physicians.

### Physical activity assessment

Physical activity levels were assessed using the self-administered LASA Physical Activity Questionnaire (LAPAQ), a validated questionnaire against 7-day diary and pedometer (correlation ranges: 0.56–0.68) demonstrating good test-retest reliability (weighted kappa: 0.65–0.75).^35^ This questionnaire contains questions regarding the frequency and duration of walking, cycling, sports, gardening, and housework over the preceding two weeks. Additionally, in the questionnaire, participants could mention other sports they participated in that were not captured by the questions in LAPAQ. To evaluate the intensity of various activities, we assigned Metabolic Equivalent of Task (MET) values to each activity collected by the questionnaire, based on the 2011 revised edition of the Compendium of Physical Activities.^36^ We calculated the weekly volume of each activity (in MET-hours) by multiplying the MET values by the time spent on each activity per week. The total physical activity volume is the sum of all physical activity volumes. We defined moderate- and vigorous-level physical activity according to the 2021 guidelines for cardiovascular disease prevention in clinical practice from European Society of Cardiology,^5^ as a MET between 3 and 5.9 for moderate physical activity and a MET over 6 for vigorous physical activity. We then calculated the volume of moderate and vigorous physical activity for each participant. The moderate-vigorous activity volume was defined as the sum of moderate and vigorous physical activity.

### Carotid plaque assessment

To evaluate vulnerable plaque components—IPH and LRNC—in carotid arteries, a 1.5-T scanner from GE Healthcare, located in Milwaukee, WI, USA, was used to perform MRI. The average time between baseline carotid MRI and the baseline physical activity data collection is 0.1 years (interquartile range [IQR]: −0.6 to 1.1). MRI examinations were performed with a bilateral phased-array surface coil. The scanning process began with 2-dimensional time-of-flight MR angiography to locate the carotid bifurcations, and several high-resolution MRI sequences were obtained sequentially. A detailed description of the standardised protocol of MRI scanning and image reviewing was described previously^37^ and is provided in the Supplemental Material, along with the data on the reproducibility of image review.

### Assessment of stroke, all-cause mortality and other covariates

Stroke was defined according to the definition of the World Health Organisation.^38^ Prevalent stroke at the enrolment of the study was evaluated during an interview with a trained physician and confirmed using medical records. Subsequently, participants were continuously monitored for incident stroke through the linkage of the study database with files from general practitioners. Files from nursing homes, physicians and general practitioners of participants who moved out of the study district were also assessed. Additional information, including clinical notes and neuroimaging reports, was obtained from hospital records. Potential strokes were reviewed by research physicians and validated by an experienced stroke neurologist. For the current study, baseline stroke history was defined as stroke onset prior to the first MRI scan. Furthermore, vital status information was acquired by automatically linking general practitioner files with the study database. Municipal records of vital status were also reviewed.^39^ Participants were followed from the study baseline until incident stroke, death, last health status update indicating they were stroke-free, or January 1, 2022, whichever came first.

Data on baseline cardiovascular risk factors were gathered through interviews, physical examinations, and blood sampling.^34^ Information on age, sex, educational attainment, medication use, and occupational and smoking status (never, current, or former) was ascertained during home interview. Height and weight were measured, from which body mass index (BMI, kg/m^2^) was computed. Hypercholesterolaemia was defined as total serum cholesterol of ≥6.2 mmol/L and/or the use of lipid-lowering medication. Blood pressure was measured in a sitting position using a random-zero sphygmomanometer. Hypertension was defined as a systolic blood pressure >140 mmHg and/or a diastolic blood pressure >90 mmHg and/or the use of antihypertensive medication. Diabetes was defined as a fasting serum glucose level ≥7.0 mmol/L and/or the use of blood glucose-lowering medication. Cognitive function was assessed using the Mini-Mental State Examination (MMSE), with scores below 24 indicating cognitive impairment.

### Statistical analysis

#### Physical activity and plaque components incidence

To study the incidence of the specific plaque components, participants with the component under study at baseline were excluded. For example, when investigating the association of physical activity with incident IPH, all participants with IPH on either side of carotid arteries at baseline were excluded from the analyses. Generalised estimating equations (GEE) models with a binomial distribution and a logit link function were used to calculate the odds ratio (OR) for the association of physical activity and incident plaque components, accounting for within-subject correlation of carotid arteries and adjusting for follow-up time, sub cohort in the Rotterdam Study, sex, baseline age, follow-up time, BMI, smoking status, hypercholesterolaemia, hypertension, diabetes, education level, and baseline maximum carotid IMT. To address potential selection bias—given that only 699 of 1330 participants with baseline MRI also underwent follow-up MRI—an inverse probability of attrition weighting was applied.^40^ The attrition probability for each participant was estimated using the same covariates included into the above-mentioned GEE models with the addition of cognitive dysfunction, and the weight was calculated as the inverse of the predicted probability of having follow-up MRI. Non-linearity was assessed by comparing the quasi-likelihood under the independence model criterion (QIC) and QICu (uncorrected QIC) values between GEE models with and without natural splines (freedom of three) for physical activity, where lower values indicated better model fit. These criteria are intended for comparing GEE models that share the same correlation structure but differ in covariate specifications, such as the inclusion of linear versus nonlinear terms.^41^ In addition, we used the Wald test to present the corresponding P-value for non-linearity.

Given the potential measurement error of questionnaire-based physical activity assessments, ranking may offer greater reliability, and to explore a threshold at which risk becomes clinically meaningful, physical activity was categorised in various ways. We created sex-specific tertiles, quartiles, and quintiles for total, moderate-vigorous and moderate physical activity volume, respectively. Because most participants did not perform any vigorous-level physical activity, we defined the lowest vigorous physical activity group as persons who did not perform any vigorous physical activity, and created sex-specific median split, tertiles, and quartiles for those who did vigorous physical activity. Total and moderate-vigorous physical activity were also categorised into three groups based on previous literatures^42^^,^^43^: less than 25, 25–50, and at least 50 MET-hours/week, which are recognised as referent, high, and very high levels of overall physical activity among general populations. Physical activity was entered into regression models as a continuous or categorical variable with the lowest level of physical activity set as the reference group.

#### Physical activity and risk of stroke stratified by plaque component

Participants were recorded as positive for a plaque component if the component was identified in one or both carotid arteries. The end-point was defined as incident stroke, all-cause mortality (competing event) or end of follow-up. Cause-specific Cox models, incorporating an interaction term between baseline plaque components and physical activity, were used to assess whether plaque components modified the association between physical activity and stroke. The proportional hazards assumption was examined by plotting Schoenfeld residuals against time, and no violation of the assumption was found (results not shown). We also investigated the potential non-linear association between physical activity and stroke by modelling physical activity using natural splines with three degrees of freedom in Cox models.

Missing values of the covariates were imputed using multiple imputations (m = 30) with 30 iterations each, and the coefficients of the regression models were pooled from 30 complete imputed datasets. The effect of sex was tested by including a sex-by-physical activity interaction term in all aforementioned regression models, which was not statistically significant (P for interaction: 0.13–0.99, results not shown). In a sensitivity analysis, we repeated all regression analyses after excluding participants employed at baseline, as occupational physical activity was not collected in the used physical activity questionnaire. The authors had full access to the database population used to create the study population. The analyses were done using R software (R 4.2.1; R Foundation for Statistical Computing). The P-value threshold used for significance was 0.05 and all tests were two-sided.

### Role of funders

The funders had no role in study design, data collection, data analyses, interpretation, or writing of report.

## Results

### Baseline characteristics

1330 participants underwent baseline carotid MRI, with a mean age of 71.5 years (SD: 8.9), and 45.7% were women (Table 1). Median volumes were 34.2 (IQR: 14, 78), 25.7 (IQR: 12.0, 52.0), 21.6 (IQR: 10.8, 42.0), and 6.6 (IQR: 0, 5.3) MET-hours/week for total, moderate-to-vigorous, moderate-level, and vigorous-level physical activity, respectively. Compared to the lowest physical activity group, participants in the highest physical activity group had a more favourable anthropometric and cardiovascular risk profile at baseline, such as a lower mean BMI and a lower prevalence of hypertension and diabetes (Supplemental Tables S1–S4). 699 participants had an eligible follow-up carotid MRI, with a median interval of 5.9 years (IQR: 5.6–6.8 years) between the baseline and follow-up MRI, who were on average younger, had a more favourable cardiovascular risk profile, and showed a lower prevalence of cognitive dysfunction compared to those with only a baseline MRI (Table 1). Men and women had similar baseline age and BMI; however, women exhibited a more favourable cardiovascular risk profile than men (Supplemental Table S5).

**Table 1:** Baseline characteristics of participants.

	Total population	With second MRI	Without second MRI
*N*, person	1330	699	631
Woman	607 (45.6%)	302 (43.2%)	307 (48.3%)
Baseline age, years	71.5 (8.8)	68.4 (7.9)	74.8 (8.6)
Body mass index	27.2 (3.7)	27.1 (3.4)	27.2 (3.9)
Smoking status			
Never smoking	368 (27.7%)	200 (28.6%)	168 (26.6%)
Current smoking	202 (15.2%)	97 (13.9%)	105 (16.6%)
Former smoking	760 (57.1%)	402 (57.5%)	358 (56.7%)
Higher education	264 (19.8%)	166 (23.7%)	98 (15.5%)
Hypercholesterolaemia	777 (58.4%)	408 (58.4%)	372 (58.6%)
Hypertension	1070 (80.5%)	534 (76.4%)	539 (84.9%)
Diabetes	238 (17.9%)	119 (17.0%)	120 (18.9%)
Cognitive dysfunction	56 (4.2%)	14 (2.0%)	42 (6.6%)
Total physical activity, MET-h/week	34.5 [14.2, 77.5]	41.3 [17.6, 83.3]	27.9 [11.3, 70.5]
Moderate-to-vigorous physical activity, MET-h/week	25.7 [12.0, 52.0]	30.3 [14.4, 54.8]	21.0 [9.0, 46.6]
Moderate physical activity, MET-h/week	21.7 [10.8, 42]	25.2 [12.9, 45]	19.5 [8.5, 39.1]
Vigorous physical activity, MET-h/week	0 [0, 5.4]	0 [0, 8.3]	0 [0, 3.2]
*N*, plaque	2445	1271	1174
Maximum IMT, mm	3.19 (0.91)	3.15 (0.84)	3.24 (0.97)
Baseline IPH	518 (21.2%)	209 (16.4%)	309 (26.3%)
Baseline LRNC	748 (30.6%)	398 (31.3%)	350 (29.8%)

Values are *n* (%) or mean (SD) or median [interquartile range] as applicable.

IMT, intima-media thickness; IPH, intraplaque haemorrhage; LRNC, lipid-rich necrotic core; METh, metabolic equivalent task∗hours.

Higher education (higher vocational education or university), hypercholesterolaemia (total cholesterol ≥6.2 mmol/L and/or using lipid-reducing drug), diabetes (fasting glucose ≥7.0 mmol/L and/or using anti-diabetes drug and/or self-reported diabetes history), hypertension (systolic blood pressure ≥140 mmHg and/or diastolic blood pressure ≥90 and/or using antihypertensive drug). Cognitive dysfunction (Mini Mental State Examination score <24).

### Physical activity and incidence of plaque components

As shown in Table 2, higher volumes of moderate-to-vigorous and vigorous physical activity were associated with an increased risk of incident IPH, with adjusted OR per 20 MET-hours/week increase of 1.10 (95% CI: 1.01, 1.20; P = 0.045) and 1.25 (95% CI: 1.07, 1.47; P = 0.005), respectively. The association between total physical activity and IPH was borderline significant (adjusted OR: 1.08 [95% CI: 0.99, 1.17; P = 0.072]). Similar patterns were observed for incident LRNC, with adjusted betas of 1.08 (95% CI: 1.01, 1.15; P = 0.044), 1.09 (95% CI: 1.01, 1.19; P = 0.049), and 1.35 (95% CI: 1.10, 1.65; P = 0.004) for total, moderate-to-vigorous, and vigorous physical activity, respectively. No nonlinear relationship was found between physical activity and incident components (Supplemental Table S6).

**Table 2:** Associations of physical activity with incident carotid IPH and LRNC.

Per 20 MET-h/week increase	Incident IPH 147 incidence/937 at risk		Incident LRNC 298 incidence/677 at risk	
	Odds ratio (95% CI)	P	Odds ratio (95% CI)	P
Total physical activity	1.08 (0.99, 1.17)	0.072	1.08 (1.01, 1.15)	0.044
Moderate to vigorous physical activity	1.10 (1.01, 1.20)	0.045	1.09 (1.01, 1.19)	0.049
Moderate physical activity	1.05 (0.94, 1.18)	0.409	1.03 (0.93, 1.15)	0.573
Vigorous physical activity	1.25 (1.07, 1.47)	0.005	1.35 (1.10, 1.65)	0.004

IPH, intraplaque haemorrhage; LRNC, lipid-rich necrotic core; CI, confidence interval; MET, metabolic equivalent task.

Physical activity was entered into the model as a continuous variable with a unit of 20 MET-h/week. Odds ratios were estimated using generalised estimated equations with a binomial distribution and a logit link function, adjusting for the follow-up time between two MRI measurements, sex, baseline age, body mass index, smoking status, Rotterdam Study sub-cohort, educational level, hypercholesterolaemia, hypertension, diabetes, and maximum intima-media thickness.

**Fig. 2:**
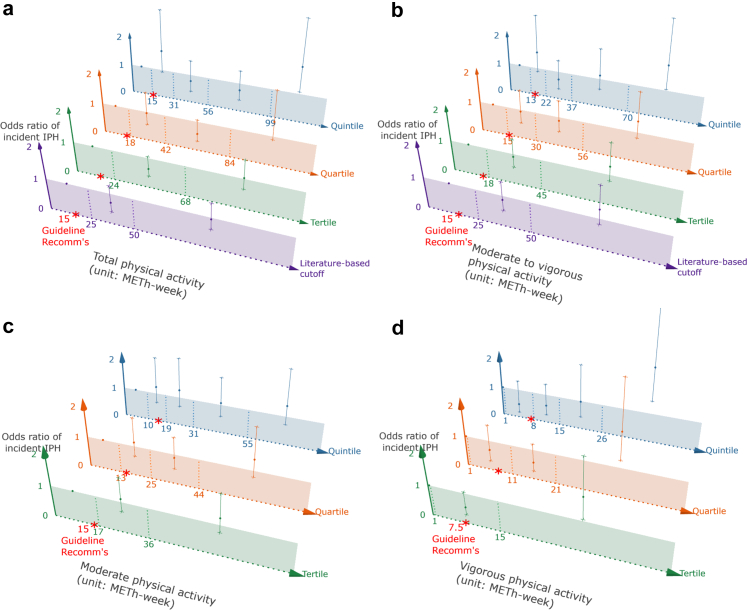
**The association between physical activity and incident intraplaque haemorrhage (N = 699)**. IPH, intraplaque haemorrhage; MET-h-week, metabolic equivalent task hours per week. Figure 2 demonstrates the associations between different levels of physical activity (total [2a], moderate to vigorous [2b], moderate [2c], and vigorous [2d]) at different cut-off values and the risk of developing IPH. The literature-based cut-off values of high volume for total and moderate to vigorous physical activity were based on the Physical Activity Guidelines Advisory Committee Report, as adapted by previous studies (Laura et al., JAMA Cardiology, 2019; Kerem et al., JAMA Cardiology, 2024). Sex-specific tertiles, quartiles, and quintiles are applied for total, moderate to vigorous, and moderate physical activity. For vigorous physical activity, because most participants did not engage in any vigorous physical activity, the lowest group was defined as individuals who reported none, and sex-specific median split, tertiles, and quartiles were created among those who did engage in vigorous physical activity. The recommended physical activity levels were adapted from cardiovascular disease primary prevention guidelines, which recommends at least 150 min of moderate and/or 75 min of vigorous activity per week—equivalent to approximately 15, 15, 15, and 7.5 MET-hours/week for total, moderate-to-vigorous, moderate, and vigorous activity, respectively. Odds ratios were estimated using generalised estimated equation with a binomial distribution and a logit link function, adjusting for the follow-up time between two MRI measurements, sex, baseline age, body mass index, smoking status, Rotterdam Study sub-cohort, educational level, hypercholesterolaemia, hypertension, diabetes, and maximum intima-media thickness.

**Fig. 3:**
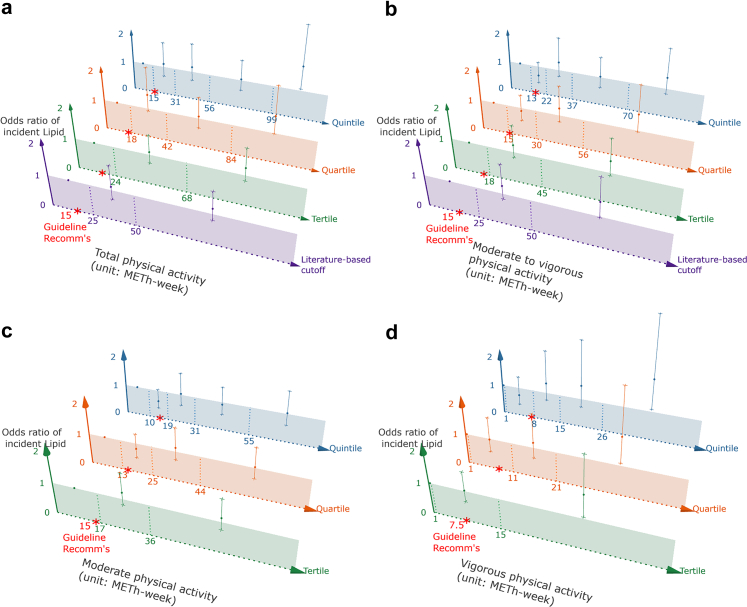
**The association between physical activity and incident lipid-rich necrotic core (N = 699)**. LRNC, lipid-rich necrotic core; MET-h-week, metabolic equivalent task hours per week. Figure 3 demonstrates the associations between different levels of physical activity (total [3a], moderate to vigorous [3b], moderate [3c], and vigorous [3d]) at different cut-off values and the risk of developing LRNC. The literature-based cut-off values of high volume for total and moderate to vigorous physical activity were based on the Physical Activity Guidelines Advisory Committee Report, as adapted by previous studies (Laura et al., JAMA Cardiology, 2019; Kerem et al., JAMA Cardiology, 2024). Sex-specific tertiles, quartiles, and quintiles are applied for total, moderate to vigorous, and moderate physical activity. For vigorous physical activity, because most participants did not engage in any vigorous physical activity, the lowest group was defined as individuals who reported none, and sex-specific median split, tertiles, and quartiles were created among those who did engage in vigorous physical activity. The recommended physical activity levels were adapted from cardiovascular disease primary prevention guidelines, which recommends at least 150 min of moderate and/or 75 min of vigorous activity per week—equivalent to approximately 15, 15, 15, and 7.5 MET-hours/week for total, moderate-to-vigorous, moderate, and vigorous activity, respectively. Odds ratios were estimated using generalised estimated equation with a binomial distribution and a logit link function, adjusting for the follow-up time between two MRI measurements, sex, baseline age, body mass index, smoking status, Rotterdam Study sub-cohort, educational level, hypercholesterolaemia, hypertension, diabetes, and maximum intima-media thickness.

To explore the potential threshold of physical activity volume at which elevated risk becomes clinically meaningful, different categorisations were performed for physical activity, with the lowest physical activity volume as the reference group for comparisons. In Fig. 2 and Supplemental Table S7, for incident IPH risk, statistically significant elevations were observed in the highest quintile of total and moderate-to-vigorous physical activity (adjusted OR: 1.93 [95% CI: 1.07, 3.47], cut-off: 96 MET-hours/week for total; adjusted OR: 1.87 [95% CI: 1.04, 3.37], cut-off: 68 MET-hours/week for moderate-to-vigorous), as well as in the highest tertile, quartile, and quintile of vigorous physical activity (adjusted OR range: 1.75–2.54, all P < 0.05; cut-off range: 15–26 MET-hours/week). In Fig. 3 and Supplemental Table S8, for incident LRNC risk, significant elevations were observed in the highest quintile of total physical activity (adjusted OR: 1.84 [95% CI: 1.06, 3.18], cut-off: 96 MET-hours/week) and the highest tertile, quartile, and quintile of vigorous physical activity (adjusted OR range: 1.75–2.10, all P < 0.05). The thresholds associated with an increased risk of incident components substantially exceed the physical activity levels recommended in cardiovascular disease prevention guidelines, which recommend more than 150 min of moderate or 75 min of vigorous activity per week—equivalent to approximately 15, 15, and 7.5 MET-hours/week for total, moderate-to-vigorous, and vigorous activity, respectively. No association was found for moderate-level physical activity. Comparable results were obtained after excluding participants in paid employment at baseline (19% [135 out of 699], Supplemental Tables S9–S11).

### Physical activity and risk of stroke stratified by plaque component

Among 1330 participants free of stroke at baseline, 127 participants experienced a stroke during a median follow-up time of 9.5 years (IQR: 8.2 years–11.8 years). No non-linear relationship was found for the association between physical activity and the risk of first-ever stroke (all P for nonlinearity >0.05). Effect modification by the presence of vulnerable plaque components at baseline (either IPH or LRNC) was observed in the association of physical activity with first-ever stroke (P for interaction = 0.010 for total; P for interaction = 0.095 for moderate-to-vigorous, Table 3). Among participants free of vulnerable plaque components, higher volumes of physical activity were associated with a lower risk of first-ever stroke (adjusted hazard ratio [HR]: 0.69 [95% CI: 0.54–0.89] for total; 0.71 [95% CI: 0.53–0.96] for moderate-to-vigorous). Although the associations for moderate and vigorous activity individually were not statistically significant, the effect sizes were comparable (adjusted HR range: 0.47–0.73). However, no association was observed in those with vulnerable plaque components at baseline (adjusted HR range: 0.98–1.00). Excluding employed participants at baseline did not change the effect modification of vulnerable plaque components on the association between physical activity and stroke (14% [182 out of 1330], Supplemental Table S12).

**Table 3:** Hazards for first-ever stroke by the presence of vulnerable carotid plaque components.

Per 20 MET-h/week	Full cohort 127 incident cases/1330 at risk			Subgroup without vulnerable components 38 incident cases/567 at risk	Subgroup with vulnerable components 89 incident cases/763 at risk
	Hazards ratio (95% CI)	P nonlinear	P interaction	Hazards ratio (95% CI)	Hazards ratio (95% CI)
Total physical activity	0.95 (0.87, 1.04)	0.595	0.010	0.69 (0.54, 0.89)	1.00 (0.91, 1.10)
Moderate to vigorous physical activity	0.94 (0.84, 1.05)	0.764	0.095	0.71 (0.53, 0.96)	0.98 (0.88, 1.11)
Moderate physical activity	0.94 (0.83, 1.07)	0.734	0.166	0.73 (0.52, 1.02)	0.98 (0.86, 1.12)
Vigorous physical activity	0.87 (0.65, 1.17)	0.687	0.209	0.47 (0.17, 1.31)	0.98 (0.72, 1.34)

CI, confidence interval; MET, metabolic equivalent task.

Physical activity was entered into the model as a continuous variable with a unit of 20 MET-h/week. Hazards ratios were estimated using cause-specific Cox models, adjusting for sex, baseline age, body mass index, smoking status, RS-cohort index, education, hypercholesterolaemia, hypertension, diabetes, maximum intima-media thickness. *P* for interaction was obtained by including an interaction term of physical activity and IPH in the model. The median of follow-up time is 9.5 years (inter-quartile range: 8.2 years, 11.8 years).

The presence of vulnerable components was defined as the presence of intraplaque haemorrhage or/and lipid-rich necrotic core.

## Discussion

Among community-dwelling older adults with subclinical carotid atherosclerotic plaques, higher volumes of total, moderate-to-vigorous, and vigorous physical activity were associated with an increased risk of incident IPH and LRNC, particularly for those in the highest quartile or quintile of physical activity volume—levels that substantially exceed the minimum recommendation in current cardiovascular disease prevention guidelines. No association was found between moderate-level physical activity and vulnerable plaque components. Furthermore, the association between physical activity and first-ever stroke differed by the presence of vulnerable carotid plaques, with beneficial effects observed only among participants without IPH or LRNC.

IPH, characterised by bleeding within an atherosclerotic plaque due to the rupture or leakage of neo-vessels, and LRNC, a hallmark of advanced plaques composed mainly of extracellular lipids and cellular debris, are key features of plaque vulnerability.^44^ Both IPH and LRNC have been demonstrated to be closely related to subsequent atherosclerotic CVD,^29^^,^^31^^,^^32^ and particularly, IPH is regarded as a stronger predictor for subsequent stroke than any other known clinical risk factor.^45^ Two previous studies have examined the cross-sectional relationship between physical activity and carotid vulnerable components. In the Atherosclerosis Risk in Communities Study, Kumar et al. reported no association between physical activity and LRNC.^46^ Mury et al. observed that IPH was less frequent when comparing the most active group (48%) with the inactive group (74%) in a small histological study (N = 90).^47^ Several factors may contribute to this discrepancy between earlier findings and the present study: First, previous studies were conducted in cross-sectional settings, limiting the ability to establish temporal relationships; Second, the volume of physical activity in the most active group in our study—where an increased risk of vulnerable plaque components was observed—was substantially higher (over 90 MET-h/week of total physical activity) than that reported in previous studies, which ranged from 15 to 24 MET-h/week.

In recent years, there has been a debate surrounding the curvilinear association of physical activity with CVD and mortality,^10^^,^^13^^,^^14^^,^^48^, ^49^, ^50^, ^51^ suggesting that the benefits of physical activity may diminish above a certain threshold. The evidence on high-volume physical activity and atherosclerosis largely comes from studies of coronary arteries in athletes, as summarised in the latest review by Guido et al., which highlights that high physical activity may even accelerate the development of atherosclerosis.^21^, ^22^, ^23^^,^^42^^,^^52^ Our results extend these findings by showing that in community-dwelling older individuals with subclinical carotid atherosclerosis, higher volumes of physical activity were associated with the development of vulnerable plaque components. Vincent et al. discovered that very vigorous-intensity exercise, rather than high-volume exercise, was associated with a greater progression of coronary atherosclerosis.^23^ In this study, we found no association of moderate level physical activity with IPH or LRNC, supporting the hypothesis that an excessive volume of vigorous level activity, rather than moderate level, may adversely affect plaque vulnerability. Nonetheless, this study does not contradict the benefits of vigorous physical activity in cardiac rehabilitation. A recent randomised controlled trial demonstrated that high-intensity interval training (<10 MET-hours weekly) safely and effectively improved cardiorespiratory fitness in coronary artery disease patients.^53^ In the present study, the threshold for increased risk of vulnerable plaque components was 26 MET-hours per week of vigorous activity, which is substantially higher than the maximum volume used in previous studies.

The mechanisms through which physical activity adversely influences atherosclerosis remain unclear. A large volume of physical activity may accelerate the progression of coronary atherosclerosis by influencing haemodynamic parameters, immune system, and calcium homoeostasis.^52^ For carotid arteries, the presence of plaque may particularly lead to adverse haemodynamic responses of physical activity on plaque vulnerability. Elevated systolic blood pressure is a key risk factor for carotid vulnerable components, especially IPH.^26^ During submaximal exercise, systolic blood pressure often exceeds 160 mmHg in older persons,^54^ while diastolic blood pressure remains relatively stable, resulting in a significant increase in pulse pressure—another independent risk factor for IPH.^55^ Additionally, vigorous exercise can induce turbulent blood flow at the sites with carotid plaque,^56^^,^^57^ causing endothelial dysfunction and lipid retention,^58^ which may increase the risk of LRNC or IPH.^59^

In this study, the favourable association of physical activity with stroke was only found in persons without vulnerable plaques. Previous studies have indicated that the beneficial effects of physical activity are more pronounced among individuals with low strength^60^ and with CVD.^61^ Our study, on the other hand, underscores the importance of identifying persons who appear healthy but may deserve extra attention for the potential adverse effects of physical activity. Another noteworthy finding is that although we observed excessively high physical activity is associated with developing vulnerable carotid plaque, this does not translate to a higher risk of subsequent stroke. One possible explanation could be that persons in the highest physical activity group had a more favourable anthropometric and cardiovascular risk profile, resulting in a lower average risk of stroke, and even if some individuals in this group have a higher risk of vulnerable carotid plaque, the overall risk of stroke remains lower.

### Strengths and limitations

The strengths of our study include a prospective study design, community-dwelling participants and repeated carotid MRIs of plaque components. These elements provide a clear temporality and good generalisability to community-dwelling older adults with subclinical atherosclerosis. The limitations of this study need to be discussed. First, participants’ physical activity was based on self-report, which has been shown to moderately correlate with objective measurement using pedometer (correlation coefficient = 0.56, P < 0.001).^35^ As a remedy, different categorisations of physical activity were applied. LAPAQ has been shown to be reliable in ranking older adults in the Netherlands by their levels of physical activity, with a gross misclassification rate of 11% compared to objective measures using pedometer.^35^ Therefore, it is less likely to misclassify physically inactive individuals into the group with the highest activity levels. Second, despite our best efforts to follow up with all participants, approximately 23% (233 out of 988) of those eligible for the follow-up MRI did not undergo the second carotid MRI due to financial restrictions and other unknown reasons, which may lead to a selection bias for the analysis of the change of plaque components. Although inverse probability of attrition weighting was applied in the regression models, caution is warranted when extrapolating the results, as participants who completed the follow-up MRI were healthier than the general population with atherosclerosis. Third, occupational physical activity was not collected in our study. Nonetheless, our sensitivity analyses showed comparable estimates after excluding participants who were employed at baseline. Fourth, the number of stroke cases in the subgroup with vulnerable plaque components was limited (89 events among 763 individuals at risk), resulting in insufficient power to detect a weak yet potentially protective effect of physical activity. Larger studies are needed to confirm whether physical activity reduces stroke risk in individuals with vulnerable plaques. Finally, the results were obtained in a predominantly white population, which may limit generalisability to populations of different ethnic origins.

### Conclusion

Among middle-aged and older adults with subclinical carotid atherosclerosis, excessive physical activity, particularly vigorous physical activity, is associated with a higher incidence of vulnerable carotid plaque components. The association of physical activity with stroke differs by the presence of vulnerable carotid plaque, with a beneficial effect only among those free of vulnerable carotid plaque.

## Contributors

Conceptualisation: LZ, TV, MKI, DB; Methodology: LZ, MK, TV, MKI, DB; Investigation: LZ, JO; Visualisation: LZ, JO; Funding acquisition: DB; Project administration: DB; Supervision: TV, MK, DB; Writing-original draft: LZ, JO; Writing-review & editing: LZ, MK, JO, TV, MKI, DB. All authors read and approved the final version of the manuscript. LZ, MK, and DB have accessed and verified the underlying data within the Rotterdam Study used in the manuscript.

## Data sharing statement

Data can be obtained upon request. Requests should be directed towards the management team of the Rotterdam Study (datamanagement.ergo@erasmusmc.nl), which has a protocol for approving data requests. Because of restrictions based on privacy regulations and informed consent of the participants, data cannot be made freely available in a public repository. All analysis scripts used in this study are publicly available at: https://github.com/zuolshy/SCI_coding/releases/tag/v1.0.

## Declaration of interests

Trudy Voortman has received grants or contracts from Erasmus MC, Erasmus University, Delft University, the European Union, Horizon 2020, and the Dutch Heart Foundation, and holds unpaid leadership or fiduciary roles in the Dutch Academy of Nutrition Sciences and the American Society for Nutrition. None of the other authors report any conflicts of interest related to this study.
