# Supplementary material for: Physical activity relates to carotid plaque vulnerability in older persons with subclinical carotid atherosclerosis
Source: eBioMedicine. 2025 Aug 20;119:105894. doi: 10.1016/j.ebiom.2025.105894 (PMC12396483; doi:10.1016/j.ebiom.2025.105894)
Supplement: Supplemental Materials [file mmc1.docx]

**Physical activity relates to carotid plaque vulnerability in older persons with subclinical carotid atherosclerosis**

**Brief title: Physical activity, carotid plaque vulnerability and stroke**

Luoshiyuan Zuo MSc^a^, Maryam Kavousi MD, PhD^a^, Julie A.E. van Oortmerssen MD^a^, Trudy Voortman PhD^a,b^, M Kamran Ikram MD, PhD^a,c^, Daniel BosMD, PhD^a,d^

^a^ Department of Epidemiology, Erasmus MC, University Medical Centre Rotterdam, Rotterdam, The Netherlands

^b^ Meta-Research Innovation Centre at Stanford (METRICS), Stanford University, Stanford, USA

^c^ Department of Neurology, Erasmus MC, University Medical Centre Rotterdam, Rotterdam, The Netherlands

^d^ Department of Radiology and Nuclear Medicine, Erasmus MC, University Medical Centre Rotterdam, Rotterdam, The Netherlands

**Supplemental material contents:**

- **Supplemental Method**

Technical description of MRI acquisition and image reviewing

Reproducibility of imaging reviewing

- **Supplemental Tables and Figures**

**Supplemental Table 1.** Baseline characteristics by quintiles of total physical activity

**Supplemental Table 2.** Baseline characteristics by quintiles of moderate to vigorous physical activity

**Supplemental Table 3.** Baseline characteristics by quintiles of moderate physical activity

**Supplemental Table 4.** Baseline characteristics by five divisions of vigorous physical activity

**Supplemental Table 5.** Baseline characteristics of participants

**Supplemental Table 6.** Comparison of GEE models evaluating the association between physical activity and incident carotid IPH and LRNC, with and without nonlinear terms

**Supplemental Table 7.** The association between physical activity and incident intraplaque haemorrhage

**Supplemental Table 8.** The association between physical activity and incident lipid-rich necrotic core

**Supplemental Table 9.** Associations of physical activity with incident carotid IPH and LRNC after excluding employed participants (n = 564)

**Supplemental Table 10.** The association between physical activity and incident intraplaque haemorrhage after excluding employed participants (n = 564)

**Supplemental Table 11.** The association between physical activity and incident lipid-rich necrotic core after excluding employed participants (n = 564)

**Supplemental Table 12.** Hazards for first-ever stroke by the presence of vulnerable carotid plaque components after excluding employed participants (n = 1148)

Supplemental method

**Technical description of MRI acquisition and image reviewing**

|  | 2-D | | | | 3-D^*^ | |
| --- | --- | --- | --- | --- | --- | --- |
|  | FSE-BB | | EPI | | PC-MRA | GRE |
|  | PDw | | PDw | T2w |  | T1w |
|  | Thin slice | High Resolution |  |  |  |  |
| TE, (ms) | 9.8 | 12.7 | 24.3 | 60 | 4.3 | 1.8 |
| TR, (ms) | 4800 | 2000 | 12000 | 12000 | 13 | 15.7 |
| ETL | 6 | 4 | - | - | - | - |
| Field of View, (cm) | 13x13 | 13x13 | 13x7 | 13x7 | 18x18 | 18x18 |
| Matrix | 160x128 | 224x160 | 160x160 | 160x160 | 256x128 | 192x180 |
| Slice thickness, (mm) | 0.9 | 1.2 | 1.2 | 1.2 | 1.0/0.5^†^ | 1.0/0.5^†^ |
| No. of slices | 51 | 19 | 41 | 41 | 26/52 | 124/248 |
| NEX | 2 | 3 | 20 | 25 | 1 | 1 |
| Scan time, (min.sec) | 3.36 | 4.04 | 4.00 | 5.00 | 6.13 | 6.02 |

FSE-BB indicates Fast Spin Echo Black Blood; EPI, Echo Planar Imaging; PC-MRA, Phased-Contrast Magnetic Resonance Angiography; GRE, Gradient Recalled Echo; PD, proton density; TR, repetition time; TE, echo time; ETL, echo train length; NEX, No. of excitations; 2-D, two-dimensional; 3-D, three-dimensional.

* = Axial images are reconstructed from the 3-D volume

† = Images are interpolated from 1.0 mm to 0.5 mm

Magnetic resonance imaging of the carotid arteries was performed on a 1.5 T MR scanner (GE Healthcare, Milwaukee, WI, USA) with a bilateral phased-array surface coil (Machnet, Eelde, The Netherlands). Participants were stabilised in a custom-designed head holder to reduce motion artefacts. High-resolution images were obtained using a standardised protocol. First, both carotid bifurcations were identified by means of two-dimensional (2D) time-of-flight MR angiography. Thereafter, high-resolution MRI sequences were planned to image the carotid bifurcations on both sides: four sequences in the axial plane: a proton density weighted (PDw)-fast spin echo (FSE)-black blood (BB) sequence; a PDw-FSE-BB with an increased in-plane resolution; a PDw-echo planar imaging (EPI) sequence, and a T2w-EPI sequence; and two 3D sequences: a 3D-T1w-gradient echo (GRE) sequence; and a 3D phased-contrast MR angiography ^1^.

IPH was characterised by a hyperintense region within the plaque on 3-dimensional T1-weighted gradient echo images. Calcification was defined as a hypointense region within the plaque across all imaging sequences, most prominent in the phase-contrast MR angiography. LRNC was considered present when a hypointense region was observed within the plaque, distinct from IPH or calcification in the proton density-weighted fast spin echo, proton density-weighted echo planar imaging, and T2-weighted echo planar imaging images, or when there was a relative signal intensity reduction in the T2-weighted echo planar imaging images compared to the proton density-weighted echo planar imaging images ^1^. In the fast spin echo images, the maximum plaque thickness was measured to indicate plaque size.

**Reproducibility of imaging reviewing**

The inter-reader agreement between readers for baseline and follow-up MRI scans was measured by randomly selecting the MRI scans of 49 participants (88 plaques) from the baseline scans. The inter-reader Cohen’s kappa statistics were 0.95 ± 0.04 for IPH (98% agreement) and 0.41 ± 0.10 for LRNC (72% agreement). The intra-reader agreement for follow-up MRI scans was measured by randomly selecting 20 participants (40 plaques) from the follow-up scans. These scans were read two times by the same reader who was unaware of scans’ ID, with a time interval of 2 months. The intra-reader Cohen’s kappa statistics were 0.91 ± 0.09 for IPH (98% agreement) and 0.80 ± 0.09 for LRNC (90% agreement).

Carotid intima-media thickness (IMT) was assessed using two modalities. First, in all participants of the Rotterdam Study, carotid ultrasonography was performed at baseline and follow-up visits, identifying 2,666 individuals with increased IMT who were invited for MRI. This method showed high reproducibility, with mean differences between repeated measurements of –0.004 mm (SD: 0.10) for sonographers, 0.066 mm (SD: 0.07) for readers, and –0.013 mm (SD: 0.13) between visits. Second, among 1,740 participants with baseline carotid MRI, maximum IMT was measured on high-resolution fast spin-echo sequences, with an inter-reader mean difference of 0.1 mm (SD: 0.5), indicating acceptable reproducibility.

**References:**

1. van den Bouwhuijsen QJ, Vernooij MW, Hofman A, et al. Determinants of magnetic resonance imaging detected carotid plaque components: the Rotterdam Study. *Eur Heart J* 2012;33(2):221-9. doi: 10.1093/eurheartj/ehr227

**Supplemental Table 1.** Baseline characteristics by quintiles of total physical activity

|  | Quintile 1 | Quintile 2 | Quintile 3 | Quintile 4 | Quintile 5 |
| --- | --- | --- | --- | --- | --- |
| *N*, person | 157 | 143 | 134 | 133 | 132 |
| Woman | 66 (42.0%) | 62 (43.4%) | 57 (42.5%) | 58 (43.6%) | 59 (44.7%) |
| Baseline age, years | 68.7 (8.9) | 69.4 (8.5) | 68.0 (8.1) | 68.5 (6.9) | 67.4 (6.8) |
| Body mass index | 27.2 (3.9) | 27.2 (3.3) | 27.1 (3.6) | 27.1 (3.2) | 26.7 (2.9) |
| Smoking status |  |  |  |  |  |
| Never smoking | 39 (24.8%) | 27 (18.9%) | 44 (32.8%) | 46 (34.6%) | 44 (33.3%) |
| Current smoking | 27 (17.2%) | 27 (18.9%) | 12 (9.0%) | 13 (9.8%) | 18 (13.6%) |
| Former smoking | 91 (58.0%) | 89 (62.2%) | 78 (58.2%) | 74 (55.6%) | 70 (53.0%) |
| Higher education | 28 (17.8%) | 39 (27.3%) | 43 (32.1%) | 28 (21.1%) | 27 (20.5%) |
| Hypercholesterolaemia | 89 (56.7%) | 83 (58.0%) | 72 (53.7%) | 79 (59.4%) | 87 (65.9%) |
| Hypertension | 126 (80.3%) | 115 (80.4%) | 99 (73.9%) | 103 (77.4%) | 95 (72.0%) |
| Diabetes | 32 (20.4%) | 32 (22.4%) | 21 (15.7%) | 21 (15.8%) | 14 (10.6%) |
| *N*, plaque | 283 | 263 | 241 | 243 | 241 |
| Maximum cIMT, mm | 3.09 (0.71) | 3.20 (0.82) | 3.18 (0.98) | 3.17 (0.81) | 3.10 (0.88) |
| Baseline IPH | 35 (12.4%) | 49 (18.6%) | 37 (15.4%) | 42 (17.3%) | 46 (19.1%) |
| Baseline LRNC | 73 (25.8%) | 78 (29.7%) | 80 (33.2%) | 74 (30.5%) | 93 (38.6%) |

Values are n (%) or mean (SD) or median [interquartile range] as applicable.

cIMT = carotid intima-media thickness, LRNC = lipid-rich necrotic core, IPH = intraplaque haemorrhage, Met = metabolic equivalent task.

Higher education (higher vocational education or university), hypercholesterolemia (total cholesterol >= 6.2 mmol/L and/or using lipid-reducing drug), diabetes (fasting glucose >= 7.0 mmol/L and/or using anti-diabetes drug and/or self-reported diabetes history), hypertension (systolic blood pressure >= 140 mmHg and/or diastolic blood pressure >= 90 and/or using antihypertensive drug).

**Supplemental Table 2.** Baseline characteristics by quintiles of moderate to vigorous physical activity

|  | Quintile 1 | Quintile 2 | Quintile 3 | Quintile 4 | Quintile 5 |
| --- | --- | --- | --- | --- | --- |
| *N*, person | 156 | 141 | 134 | 136 | 132 |
| Woman | 68 (43.6%) | 57 (40.4%) | 58 (43.3%) | 60 (44.1%) | 59 (44.7%) |
| Baseline age, years | 69.5 (9.1) | 69.6 (8.4) | 67.9 (7.7) | 67.8 (7.3) | 67.1 (6.4) |
| Body mass index | 27.0 (3.74) | 27.6 (3.47) | 27.2 (3.37) | 26.9 (3.36) | 26.6 (3.13) |
| Smoking status |  |  |  |  |  |
| Never smoking | 41 (26.3%) | 28 (19.9%) | 42 (31.3%) | 45 (33.1%) | 44 (33.3%) |
| Current smoking | 28 (17.9%) | 18 (12.8%) | 18 (13.4%) | 14 (10.3%) | 19 (14.4%) |
| Former smoking | 87 (55.8%) | 95 (67.4%) | 74 (55.2%) | 77 (56.6%) | 69 (52.3%) |
| Higher education | 29 (18.6%) | 32 (22.7%) | 49 (36.6%) | 29 (21.3%) | 26 (19.7%) |
| Hypercholesterolaemia | 89 (57.1%) | 82 (58.2%) | 77 (57.5%) | 74 (54.4%) | 88 (66.7%) |
| Hypertension | 123 (78.8%) | 116 (82.3%) | 101 (75.4%) | 102 (75.0%) | 96 (72.7%) |
| Diabetes | 32 (20.5%) | 31 (22.0%) | 28 (20.9%) | 17 (12.5%) | 12 (9.1%) |
| *N*, plaque | 286 | 261 | 241 | 243 | 240 |
| Maximum cIMT, mm | 3.12 (0.77) | 3.21 (0.81) | 3.13 (0.92) | 3.20 (0.82) | 3.09 (0.89) |
| Baseline IPH | 37 (12.9%) | 52 (19.9%) | 39 (16.2%) | 38 (15.6%) | 43 (17.9%) |
| Baseline LRNC | 75 (26.2%) | 72 (27.6%) | 82 (34.0%) | 84 (34.6%) | 85 (35.4%) |

Values are n (%) or mean (SD) or median [interquartile range] as applicable.

cIMT = carotid intima-media thickness, LRNC = lipid-rich necrotic core, IPH = intraplaque haemorrhage, Met = metabolic equivalent task.

Higher education (higher vocational education or university), hypercholesterolemia (total cholesterol >= 6.2 mmol/L and/or using lipid-reducing drug), diabetes (fasting glucose >= 7.0 mmol/L and/or using anti-diabetes drug and/or self-reported diabetes history), hypertension (systolic blood pressure >= 140 mmHg and/or diastolic blood pressure >= 90 and/or using antihypertensive drug).

**Supplemental Table 3.** Baseline characteristics by quintiles of moderate physical activity

|  | Quintile 1 | Quintile 2 | Quintile 3 | Quintile 4 | Quintile 5 |
| --- | --- | --- | --- | --- | --- |
| *N*, person | 155 | 144 | 133 | 137 | 130 |
| Woman | 66 (42.6%) | 59 (41.0%) | 58 (43.6%) | 61 (44.5%) | 58 (44.6%) |
| Baseline age, years | 69.2 (9.5) | 69.0 (8.0) | 68.3 (7.9) | 67.8 (6.9) | 67.7 (6.7) |
| Body mass index | 27.2 (3.85) | 27.5 (3.25) | 26.8 (3.59) | 26.9 (3.02) | 26.7 (3.35) |
| Smoking status |  |  |  |  |  |
| Never smoking | 48 (31.0%) | 30 (20.8%) | 38 (28.6%) | 49 (35.8%) | 35 (26.9%) |
| Current smoking | 23 (14.8%) | 19 (13.2%) | 22 (16.5%) | 12 (8.8%) | 21 (16.2%) |
| Former smoking | 84 (54.2%) | 95 (66.0%) | 73 (54.9%) | 76 (55.5%) | 74 (56.9%) |
| Higher education | 35 (22.6%) | 36 (25.0%) | 38 (28.6%) | 30 (21.9%) | 26 (20.0%) |
| Hypercholesterolaemia | 85 (54.8%) | 89 (61.8%) | 69 (51.9%) | 77 (56.2%) | 90 (69.2%) |
| Hypertension | 118 (76.1%) | 120 (83.3%) | 100 (75.2%) | 104 (75.9%) | 96 (73.8%) |
| Diabetes | 36 (23.2%) | 29 (20.1%) | 19 (14.3%) | 23 (16.8%) | 13 (10.0%) |
| *N*, plaque | 281 | 268 | 241 | 241 | 240 |
| Maximum cIMT, mm | 3.08 (0.71) | 3.19 (0.85) | 3.14 (0.87) | 3.26 (0.95) | 3.08 (0.80) |
| Baseline IPH | 37 (13.2%) | 48 (17.9%) | 44 (18.3%) | 33 (13.7%) | 47 (19.6%) |
| Baseline LRNC | 73 (26.0%) | 80 (29.9%) | 74 (30.7%) | 88 (36.5%) | 83 (34.6%) |

Values are n (%) or mean (SD) or median [interquartile range] as applicable.

cIMT = carotid intima-media thickness, LRNC = lipid-rich necrotic core, IPH = intraplaque haemorrhage, Met = metabolic equivalent task.

Higher education (higher vocational education or university), hypercholesterolemia (total cholesterol >= 6.2 mmol/L and/or using lipid-reducing drug), diabetes (fasting glucose >= 7.0 mmol/L and/or using anti-diabetes drug and/or self-reported diabetes history), hypertension (systolic blood pressure >= 140 mmHg and/or diastolic blood pressure >= 90 and/or using antihypertensive drug).

**Supplemental Table 4.** Baseline characteristics by five divisions of vigorous physical activity

|  | Lowest group | Quartile 1 | Quartile 2 | Quartile 3 | Quartile 4 |
| --- | --- | --- | --- | --- | --- |
| *N*, person | 466 | 57 | 58 | 57 | 61 |
| Woman | 198 (42.5%) | 26 (45.6%) | 26 (44.8%) | 25 (43.9%) | 27 (44.3%) |
| Baseline age, years | 69.4 (7.8) | 66.3 (9.4) | 66.6 (7.3) | 67.1 (7.7) | 66.2 (6.9) |
| Body mass index | 27.1 (3.55) | 27.4 (3.26) | 27.8 (3.23) | 26.3 (3.08) | 26.5 (3.08) |
| Smoking status |  |  |  |  |  |
| Never smoking | 111 (23.8%) | 20 (35.1%) | 17 (29.3%) | 20 (35.1%) | 32 (52.5%) |
| Current smoking | 77 (16.5%) | 6 (10.5%) | 7 (12.1%) | 3 (5.3%) | 4 (6.6%) |
| Former smoking | 278 (59.7%) | 31 (54.4%) | 34 (58.6%) | 34 (59.6%) | 25 (41.0%) |
| Higher education | 95 (20.4%) | 18 (31.6%) | 18 (31.0%) | 16 (28.1%) | 18 (29.5%) |
| Hypercholesterolaemia | 275 (59.0%) | 29 (50.9%) | 26 (44.8%) | 43 (75.4%) | 37 (60.7%) |
| Hypertension | 371 (79.6%) | 40 (70.2%) | 45 (77.6%) | 43 (75.4%) | 39 (63.9%) |
| Diabetes | 86 (18.5%) | 12 (21.1%) | 9 (15.5%) | 5 (8.8%) | 8 (13.1%) |
| *N*, plaque | 854 | 104 | 103 | 103 | 107 |
| Maximum cIMT, mm | 3.15 (0.82) | 3.11 (0.75) | 3.26 (0.94) | 3.15 (0.85) | 3.03 (0.91) |
| Baseline IPH | 144 (16.9%) | 17 (16.3%) | 16 (15.5%) | 23 (22.3%) | 9 (8.4%) |
| Baseline LRNC | 257 (30.1%) | 36 (34.6%) | 27 (26.2%) | 41 (39.8%) | 37 (34.6%) |

Values are n (%) or mean (SD) or median [interquartile range] as applicable.

cIMT = carotid intima-media thickness, LRNC = lipid-rich necrotic core, IPH = intraplaque haemorrhage, Met = metabolic equivalent task.

Higher education (higher vocational education or university), hypercholesterolemia (total cholesterol >= 6.2 mmol/L and/or using lipid-reducing drug), diabetes (fasting glucose >= 7.0 mmol/L and/or using anti-diabetes drug and/or self-reported diabetes history), hypertension (systolic blood pressure >= 140 mmHg and/or diastolic blood pressure >= 90 and/or using antihypertensive drug).

Because most participants did not engage in any vigorous physical activity, the lowest group was defined as individuals who reported none, and sex-specific quartiles were created among those who did engage in vigorous physical activity.

**Supplemental Table 5.** Baseline characteristics of participants

|  | Total population | Men | Women |
| --- | --- | --- | --- |
| *N*, person | 1330 | 723 | 607 |
| With follow-up MRI | 607 (45.6%) | 397 (54.9%) | 302 (49.8%) |
| Baseline age, years | 71.5 (8.8) | 71.1 (8.82) | 71.9 (8.91) |
| Body mass index | 27.2 (3.7) | 27.2 (3.25) | 27.1 (4.14) |
| Smoking status |  |  |  |
| Never smoking | 368 (27.7%) | 130 (18.0%) | 239 (39.4%) |
| Current smoking | 202 (15.2%) | 102 (14.1%) | 102 (16.8%) |
| Former smoking | 760 (57.1%) | 491 (67.9%) | 266 (43.8%) |
| Higher education | 264 (19.8%) | 188 (26.0%) | 72 (11.9%) |
| Hypercholesterolaemia | 777 (58.4%) | 396 (54.8%) | 381 (62.8%) |
| Hypertension | 1070 (80.5%) | 597 (82.6%) | 473 (77.9%) |
| Diabetes | 238 (17.9%) | 154 (21.3%) | 84 (13.8%) |
| Cognitive dysfunction | 56 (4.2 %) | 28 (3.9%) | 28 (4.6%) |
| Total physical activity, METh/week | 34.5 [14.2, 77.5] | 33.9 [15, 71.2] | 35.8 [13.8, 84] |
| Moderate-to-vigorous physical activity, METh/week | 25.7 [12.0, 52.0] | 27.4 [12.5, 52.6] | 22.5 [12, 50.1] |
| Moderate physical activity, METh/week | 21.7 [10.8, 42] | 22.5 [11.3, 42] | 20.5 [10.5, 42.5] |
| Vigorous physical activity, METh/week | 0 [0, 5.4] | 0 [0, 6.8] | 0 [0, 3.5] |
| *N*, plaque | 2445 | 1340 | 1105 |
| Maximum IMT, mm | 3.19 (0.91) | 3.10 [2.00, 9.60] | 2.90 [2.00, 8.50] |
| Baseline IPH | 518 (21.2%) | 349 (26.0%) | 169 (15.3%) |
| Baseline LRNC | 748 (30.6%) | 457 (34.1%) | 291 (26.3%) |

Values are *n* (%) or mean (SD) or median [interquartile range] as applicable.

IMT = intima-media thickness, IPH = intraplaque haemorrhage, LRNC = lipid-rich necrotic core, METh = metabolic equivalent task*hours.

Higher education (higher vocational education or university), hypercholesterolemia (total cholesterol >= 6.2 mmol/L and/or using lipid-reducing drug), diabetes (fasting glucose >= 7.0 mmol/L and/or using anti-diabetes drug and/or self-reported diabetes history), hypertension (systolic blood pressure >= 140 mmHg and/or diastolic blood pressure >= 90 and/or using antihypertensive drug). Cognitive dysfunction (Mini Mental State Examination score < 24).

**Supplemental Table 6.** Comparison of GEE models evaluating the association between physical activity and incident carotid IPH and LRNC, with and without nonlinear terms

|  | Incident IPH | | | |  | Incident LRNC | | | |  |
| --- | --- | --- | --- | --- | --- | --- | --- | --- | --- | --- |
|  | *Model with linear term of physical activity* | | *Model with nonlinear term of physical activity* | | *P for nonlinearity* | *Model without nonlinear term of physical activity* | | *Model with nonlinear term of physical activity* | | *P for nonlinearity* |
|  | QIC | QICu | QIC | QICu |  | QIC | QICu | QIC | QICu |  |
| Total physical activity | 797.01 | 793.24 | 797.66 | 794.45 | 0.062 | 930.94 | 927.76 | 933.58 | 929.93 | 0.503 |
| Moderate to vigorous physical activity | 797.17 | 793.58 | 800.41 | 797.07 | 0.542 | 931.21 | 927.99 | 934.86 | 931.41 | 0.681 |
| Moderate physical activity | 800.59 | 797.15 | 803.95 | 800.11 | 0.595 | 934.34 | 931.02 | 938.90 | 935.13 | 0.739 |
| Vigorous physical activity | 795.52 | 792.42 | 795.59 | 794.18 | 0.179 | 925.63 | 922.51 | 930.97 | 927.76 | 0.494 |

QIC = quasi-likelihood under the independence model information criterion, QICu = uncorrected form of QIC, GEE = generalised estimated equations, IPH = intraplaque haemorrhage, LRNC = lipid-rich necrotic core, CI = confidence interval, MET = metabolic equivalent task.

Estimates were obtained using GEE with a binomial distribution and a logit link function, adjusting for the follow-up time between two MRI measurements, sex, baseline age, body mass index, smoking status, Rotterdam Study cohort, educational level, hypercholesterolemia, hypertension, diabetes, and maximum intima-media thickness. Nonlinear associations with physical activity were modelled using natural splines with three degrees of freedom.

Comparison of GEE models was evaluated using both information criterion (QIC/QICu) and Wald tests. Lower values of QIC and QICu indicate better fit. QICu and Wald tests are specifically used to compare models with the same correlation structure but different covariate specifications, such as the inclusion of nonlinear terms. In this study, both QIC and QICu increased after introducing nonlinear terms of physical activity, and the Wald test did not show significant improvement, suggesting that a linear specification of physical activity provided a better model fit.

**Supplemental Table 7.** The association between physical activity and incident intraplaque haemorrhage

| Total physical activity | | | | | |
| --- | --- | --- | --- | --- | --- |
| Literature-based cutoff | < 25 MET-h/week | >= 25 & < 50 MET-h/week | >= 50 MET-h/week |  |  |
| *N* (incident IPH) | 340(58) | 198(23) | 399(66) |  |  |
| Odds ratio (95% CI) | Reference | 0.71 (0.4, 1.25); P = 0.236 | 0.95 (0.62, 1.45); P = 0.811 |  |  |
| Tertiles | T1 | T2 | T3 |  |  |
| *N* (incident IPH) | 325(57) | 308(34) | 304(56) |  |  |
| Odds ratio (95% CI) | Reference | 0.64 (0.39, 1.05); P = 0.076 | 1.03 (0.66, 1.6); P = 0.900 |  |  |
| Quartiles | Q1 | Q2 | Q3 | Q4 |  |
| *N* (incident IPH) | 249(38) | 231(35) | 227(25) | 230(49) |  |
| Odds ratio (95% CI) | Reference | 0.96 (0.56, 1.65); P = 0.894 | 0.58 (0.32, 1.06); P = 0.079 | 1.49 (0.89, 2.48); P = 0.129 |  |
| Quintiles | Q1 | Q2 | Q3 | Q4 | **Q5** |
| *N* (incident IPH) | 223(30) | 184(36) | 182(22) | 174(20) | **174(39)** |
| Odds ratio (95% CI) | Reference | 1.68 (0.93, 3.06); P = 0.087 | 0.78 (0.4, 1.5); P = 0.451 | 0.76 (0.4, 1.44); P = 0.400 | **1.93 (1.07, 3.47); P = 0.029** |
| Moderate to vigorous physical activity | | | | | |
| Literature-based cutoff | < 25 MET-h/week | >= 25 & < 50 MET-h/week | >= 50 MET-h/week |  |  |
| *N* (incident IPH) | 410(65) | 253(31) | 274(51) |  |  |
| Odds ratio (95% CI) | Reference | 0.87 (0.52, 1.44); P = 0.586 | 1.29 (0.82, 2.02); P = 0.272 |  |  |
| Tertiles | T1 | T2 | T3 |  |  |
| *N* (incident IPH) | 321(49) | 312(42) | 304(56) |  |  |
| Odds ratio (95% CI) | Reference | 0.91 (0.56, 1.47); P = 0.694 | 1.27 (0.8, 2.01); P = 0.309 |  |  |
| Quartiles | Q1 | Q2 | Q3 | Q4 |  |
| *N* (incident IPH) | 252(37) | 213(35) | 240(30) | 232(45) |  |
| Odds ratio (95% CI) | Reference | 0.94 (0.54, 1.65); P = 0.833 | 0.88 (0.5, 1.55); P = 0.657 | 1.37 (0.81, 2.31); P = 0.242 |  |
| Quintiles | Q1 | Q2 | Q3 | Q4 | **Q5** |
| *N* (incident IPH) | 224(31) | 177(33) | 181(18) | 181(28) | **174(37)** |
| Odds ratio (95% CI) | Reference | 1.57 (0.88, 2.82); P = 0.130 | 0.72 (0.37, 1.4); P = 0.336 | 1.13 (0.63, 2.05); P = 0.677 | **1.87 (1.04, 3.37); P = 0.037** |
| Moderate physical activity | | | | | |
| Tertiles | T1 | T2 | T3 |  |  |
| *N* (incident IPH) | 329(47) | 302(47) | 306(53) |  |  |
| Odds ratio (95% CI) | Reference | 1.1 (0.69, 1.76); P = 0.689 | 1.22 (0.76, 1.96); P = 0.417 |  |  |
| Quartiles | Q1 | Q2 | Q3 | Q4 |  |
| *N* (incident IPH) | 250(35) | 220(38) | 250(33) | 217(41) |  |
| Odds ratio (95% CI) | Reference | 1.13 (0.66, 1.93); P = 0.653 | 0.89 (0.51, 1.56); P = 0.682 | 1.37 (0.79, 2.38); P = 0.263 |  |
| Quintiles | Q1 | Q2 | Q3 | Q4 | Q5 |
| *N* (incident IPH) | 218(30) | 191(32) | 174(27) | 187(27) | 167(31) |
| Odds ratio (95% CI) | Reference | 1.23 (0.67, 2.23); P = 0.507 | 1.26 (0.68, 2.34); P = 0.465 | 1.07 (0.59, 1.94); P = 0.830 | 1.37 (0.72, 2.58); P = 0.335 |
| Vigorous physical activity | | | | | |
| Tertiles | T1 | T2 | **T3** |  |  |
| *N* (incident IPH) | 619(98) | 157(14) | **161(35)** |  |  |
| Odds ratio (95% CI) | Reference | 0.54 (0.29, 1.04); P = 0.064 | **1.75 (1.05, 2.91); P = 0.032** |  |  |
| Quartiles | Q1 | Q2 | Q3 | **Q4** |  |
| *N* (incident IPH) | 619(98) | 97(10) | 105(10) | **116(29)** |  |
| Odds ratio (95% CI) | Reference | 0.69 (0.31, 1.52); P = 0.353 | 0.57 (0.27, 1.19); P = 0.135 | **2.15 (1.24, 3.71); P = 0.006** |  |
| Quintiles | Q1 | Q2 | Q3 | Q4 | **Q5** |
| *N* (incident IPH) | 619(98) | 78(6) | 79(8) | 68(11) | **93(24)** |
| Odds ratio (95% CI) | Reference | 0.48 (0.18, 1.31); P = 0.155 | 0.61 (0.27, 1.37); P = 0.232 | 0.98 (0.43, 2.24); P = 0.959 | **2.54 (1.43, 4.52); P = 0.002** |

IPH = intraplaque haemorrhage, METh-week = metabolic equivalent task hours per week.

The literature-based cutoff values of high volume for total and moderate to vigorous physical activity were based on the Physical Activity Guidelines Advisory Committee Report, as adapted by previous studies (Laura et al., JAMA Cardiology, 2019; Kerem et al., JAMA Cardiology, 2024).

Sex-specific tertiles, quartiles, and quintiles are applied for total, moderate to vigorous, and moderate physical activity. For vigorous physical activity, because most participants did not engage in any vigorous physical activity, the lowest group was defined as individuals who reported none, and sex-specific median split, tertiles, and quartiles were created among those who did engage in vigorous physical activity.

Odds ratios were obtained using generalised estimated equation with a binomial distribution and a logit link function, adjusting for the follow-up time between two MRI measurements, sex, baseline age, body mass index, smoking status, Rotterdam Study sub-cohort, educational level, hypercholesterolemia, hypertension, diabetes, and maximum intima-media thickness.

**Supplemental Table 8.** The association between physical activity and incident lipid-rich necrotic core

| Total physical activity | | | | | |
| --- | --- | --- | --- | --- | --- |
| Literature-based cutoff | < 25 MET-h/week | >= 25 & < 50 MET-h/week | >= 50 MET-h/week |  |  |
| *N* (incident LRNC) | 260(109) | 127(58) | 290(131) |  |  |
| Odds ratio (95% CI) | Reference | 1.11 (0.7, 1.77); P = 0.648 | 1.17 (0.8, 1.7); P = 0.411 |  |  |
| Tertiles | T1 | T2 | T3 |  |  |
| *N* (incident LRNC) | 248(104) | 216(93) | 213(101) |  |  |
| Odds ratio (95% CI) | Reference | 1.09 (0.72, 1.63); P = 0.694 | 1.24 (0.82, 1.86); P = 0.302 |  |  |
| Quartiles | Q1 | Q2 | Q3 | Q4 |  |
| *N* (incident LRNC) | 188(73) | 161(77) | 178(73) | 150(75) |  |
| Odds ratio (95% CI) | Reference | 1.47 (0.92, 2.36); P = 0.108 | 1.07 (0.67, 1.7); P = 0.775 | 1.57 (0.98, 2.52); P = 0.059 |  |
| Quintiles | Q1 | Q2 | Q3 | Q4 | **Q5** |
| *N* (incident LRNC) | 172(69) | 138(58) | 118(57) | 143(59) | **106(55)** |
| Odds ratio (95% CI) | Reference | 1.11 (0.66, 1.86); P = 0.701 | 1.17 (0.69, 1.98); P = 0.552 | 1.04 (0.64, 1.7); P = 0.868 | **1.84 (1.06, 3.18); P = 0.030** |
| Moderate to vigorous physical activity | | | | | |
| Literature-based cutoff | < 25 MET-h/week | >= 25 & < 50 MET-h/week | >= 50 MET-h/week |  |  |
| *N* (incident LRNC) | 319(126) | 166(78) | 192(94) |  |  |
| Odds ratio (95% CI) | Reference | 1.25 (0.83, 1.91); P = 0.288 | 1.46 (0.97, 2.2); P = 0.071 |  |  |
| Tertiles | T1 | T2 | T3 |  |  |
| *N* (incident LRNC) | 245(97) | 219(99) | 213(102) |  |  |
| Odds ratio (95% CI) | Reference | 1.23 (0.82, 1.85); P = 0.322 | 1.38 (0.91, 2.08); P = 0.126 |  |  |
| Quartiles | Q1 | Q2 | Q3 | Q4 |  |
| *N* (incident LRNC) | 184(77) | 172(64) | 164(77) | 157(80) |  |
| Odds ratio (95% CI) | Reference | 0.9 (0.56, 1.43); P = 0.650 | 1.24 (0.78, 1.97); P = 0.358 | 1.54 (0.96, 2.47); P = 0.073 |  |
| Quintiles | Q1 | Q2 | Q3 | Q4 | Q5 |
| *N* (incident LRNC) | 170(73) | 147(53) | 118(57) | 125(58) | 117(57) |
| Odds ratio (95% CI) | Reference | 0.67 (0.4, 1.15); P = 0.148 | 1.28 (0.77, 2.13); P = 0.337 | 1.03 (0.63, 1.7); P = 0.895 | 1.39 (0.82, 2.37); P = 0.224 |
| Moderate physical activity | | | | | |
| Tertiles | T1 | T2 | T3 |  |  |
| *N* (incident LRNC) | 243(99) | 226(107) | 208(92) |  |  |
| Odds ratio (95% CI) | Reference | 1.21 (0.81, 1.81); P = 0.347 | 1.13 (0.75, 1.72); P = 0.559 |  |  |
| Quartiles | Q1 | Q2 | Q3 | Q4 |  |
| *N* (incident LRNC) | 191(83) | 166(63) | 165(83) | 155(69) |  |
| Odds ratio (95% CI) | Reference | 0.82 (0.51, 1.31); P = 0.398 | 1.13 (0.72, 1.78); P = 0.595 | 1.03 (0.64, 1.66); P = 0.893 |  |
| Quintiles | Q1 | Q2 | Q3 | Q4 | Q5 |
| *N* (incident LRNC) | 164(74) | 145(51) | 130(66) | 120(50) | 118(57) |
| Odds ratio (95% CI) | Reference | 0.62 (0.37, 1.04); P = 0.073 | 1.04 (0.62, 1.75); P = 0.881 | 0.92 (0.56, 1.52); P = 0.751 | 1.03 (0.6, 1.78); P = 0.903 |
| Vigorous physical activity | | | | | |
| Tertiles | T1 | T2 | **T3** |  |  |
| *N* (incident LRNC) | 468(197) | 112(49) | **97(52)** |  |  |
| Odds ratio (95% CI) | Reference | 1.02 (0.65, 1.61); P = 0.932 | **1.75 (1.05, 2.91); P = 0.033** |  |  |
| Quartiles | Q1 | Q2 | Q3 | **Q4** |  |
| *N* (incident LRNC) | 468(197) | 64(30) | 73(30) | **72(41)** |  |
| Odds ratio (95% CI) | Reference | 0.98 (0.56, 1.7); P = 0.936 | 1.17 (0.65, 2.1); P = 0.606 | **1.93 (1.08, 3.43); P = 0.026** |  |
| Quintiles | Q1 | Q2 | Q3 | Q4 | **Q5** |
| *N* (incident LRNC) | 468(197) | 51(20) | 61(29) | 46(23) | **51(29)** |
| Odds ratio (95% CI) | Reference | 0.74 (0.4, 1.39); P = 0.353 | 1.34 (0.74, 2.44); P = 0.339 | 1.47 (0.72, 2.98); P = 0.292 | **2.10 (1.08, 4.1); P = 0.030** |

LRNC = lipid-rich necrotic core, METh-week = metabolic equivalent task hours per week.

The literature-based cutoff values of high volume for total and moderate to vigorous physical activity were based on the Physical Activity Guidelines Advisory Committee Report, as adapted by previous studies (Laura et al., JAMA Cardiology, 2019; Kerem et al., JAMA Cardiology, 2024).

Sex-specific tertiles, quartiles, and quintiles are applied for total, moderate to vigorous, and moderate physical activity. For vigorous physical activity, because most participants did not engage in any vigorous physical activity, the lowest group was defined as individuals who reported none, and sex-specific median split, tertiles, and quartiles were created among those who did engage in vigorous physical activity.

Odds ratios were obtained using generalised estimated equation with a binomial distribution and a logit link function, adjusting for the follow-up time between two MRI measurements, sex, baseline age, body mass index, smoking status, Rotterdam Study sub-cohort, educational level, hypercholesterolemia, hypertension, diabetes, and maximum intima-media thickness.

**Supplemental Table 9.** Associations of physical activity with incident carotid IPH and LRNC after excluding employed participants (n = 564)

|  | Incident IPH | | Incident LRNC | |
| --- | --- | --- | --- | --- |
| per 20 Met-hours/week increase | *Odds ratio* (95% CI) | *P* | *Odds ratio* (95% CI) | *P* |
| Total physical activity | 1.08 (0.99, 1.17) | 0.065 | 1.10 (1.02, 1.18) | 0.016 |
| Moderate to vigorous physical activity | 1.11 (1.01, 1.22) | 0.035 | 1.11 (1.01, 1.22) | 0.038 |
| Moderate physical activity | 1.07 (0.95, 1.20) | 0.290 | 1.03 (0.92, 1.15) | 0.642 |
| Vigorous physical activity | 1.25 (1.05, 1.50) | 0.013 | 1.44 (1.17, 1.79) | 0.001 |

IPH = intraplaque haemorrhage, LRNC = lipid-rich necrotic core, CI = confidence interval, MET = metabolic equivalent task.

Odds ratios were obtained using generalised estimated equations with a binomial distribution and a logit link function, adjusting for the follow-up time between two MRI measurements, sex, baseline age, body mass index, smoking status, Rotterdam Study sub-cohort, educational level, hypercholesterolemia, hypertension, diabetes, and maximum intima-media thickness.

**Supplemental Table 10.** The association between physical activity and incident intraplaque haemorrhage after excluding employed participants (n = 564)

| Total physical activity | | | | | |
| --- | --- | --- | --- | --- | --- |
| Literature-based cutoff | < 25 MET-h/week | >= 25 & < 50 MET-h/week | >= 50 MET-h/week |  |  |
| *N* (incident IPH) | 268(51) | 140(18) | 328(55) |  |  |
| Odds ratio (95% CI) | Reference | 0.75 (0.4, 1.38); P = 0.353 | 0.9 (0.58, 1.4); P = 0.628 |  |  |
| Tertiles | T1 | T2 | T3 |  |  |
| *N* (incident IPH) | 258(50) | 221(25) | 257(49) |  |  |
| Odds ratio (95% CI) | Reference | 0.62 (0.36, 1.07); P = 0.085 | 1.02 (0.64, 1.61); P = 0.949 |  |  |
| Quartiles | Q1 | Q2 | Q3 | Q4 |  |
| *N* (incident IPH) | 190(31) | 177(34) | 176(15) | 193(44) |  |
| Odds ratio (95% CI) | Reference | 1.16 (0.66, 2.06); P = 0.601 | 0.46 (0.23, 0.92); P = 0.028 | 1.57 (0.91, 2.7); P = 0.104 |  |
| Quintiles | Q1 | Q2 | Q3 | Q4 | **Q5** |
| *N* (incident IPH) | 153(22) | 147(34) | 135(16) | 158(17) | **143(35)** |
| Odds ratio (95% CI) | Reference | 1.84 (0.98, 3.46); P = 0.057 | 0.86 (0.42, 1.75); P = 0.672 | 0.7 (0.34, 1.42); P = 0.321 | **2.01 (1.09, 3.7); P = 0.025** |
| Moderate to vigorous physical activity | | | | | |
| Literature-based cutoff | < 25 MET-h/week | >= 25 & < 50 MET-h/week | >= 50 MET-h/week |  |  |
| *N* (incident IPH) | 323(57) | 189(23) | 224(44) |  |  |
| Odds ratio (95% CI) | Reference | 0.76 (0.43, 1.34); P = 0.347 | 1.27 (0.79, 2.03); P = 0.326 |  |  |
| Tertiles | T1 | T2 | T3 |  |  |
| *N* (incident IPH) | 254(42) | 234(34) | 248(48) |  |  |
| Odds ratio (95% CI) | Reference | 0.95 (0.57, 1.59); P = 0.837 | 1.27 (0.78, 2.06); P = 0.331 |  |  |
| Quartiles | Q1 | Q2 | Q3 | Q4 |  |
| *N* (incident IPH) | 197(31) | 168(33) | 180(21) | 191(39) |  |
| Odds ratio (95% CI) | Reference | 1.14 (0.64, 2.05); P = 0.653 | 0.76 (0.4, 1.45); P = 0.404 | 1.44 (0.83, 2.5); P = 0.192 |  |
| Quintiles | Q1 | Q2 | Q3 | Q4 | **Q5** |
| *N* (incident IPH) | 161(24) | 138(32) | 142(14) | 158(24) | **137(30)** |
| Odds ratio (95% CI) | Reference | 1.68 (0.9, 3.12); P = 0.101 | 0.71 (0.35, 1.44); P = 0.342 | 1.02 (0.53, 1.94); P = 0.960 | **1.9 (1.03, 3.49); P = 0.040** |
| Moderate physical activity | | | | | |
| Tertiles | T1 | T2 | T3 |  |  |
| *N* (incident IPH) | 250(39) | 239(41) | 247(44) |  |  |
| Odds ratio (95% CI) | Reference | 1.21 (0.73, 2); P = 0.461 | 1.21 (0.73, 1.99); P = 0.465 |  |  |
| Quartiles | Q1 | Q2 | Q3 | Q4 |  |
| *N* (incident IPH) | 192(30) | 174(33) | 192(24) | 178(37) |  |
| Odds ratio (95% CI) | Reference | 1.25 (0.71, 2.22); P = 0.441 | 0.86 (0.46, 1.59); P = 0.627 | 1.44 (0.82, 2.53); P = 0.205 |  |
| Quintiles | Q1 | Q2 | Q3 | Q4 | Q5 |
| *N* (incident IPH) | 157(25) | 142(24) | 136(25) | 168(23) | 133(27) |
| Odds ratio (95% CI) | Reference | 1.17 (0.61, 2.22); P = 0.636 | 1.44 (0.76, 2.75); P = 0.268 | 0.88 (0.45, 1.69); P = 0.697 | 1.54 (0.82, 2.9); P = 0.185 |
| Vigorous physical activity | | | | | |
| Tertiles | T1 | T2 | T3 |  |  |
| *N* (incident IPH) | 499(87) | 111(9) | 126(28) |  |  |
| Odds ratio (95% CI) | Reference | 0.45 (0.21, 0.95); P = 0.036 | 1.63 (0.94, 2.82); P = 0.084 |  |  |
| Quartiles | Q1 | Q2 | Q3 | **Q4** |  |
| *N* (incident IPH) | 499(87) | 65(7) | 81(5) | **91(25)** |  |
| Odds ratio (95% CI) | Reference | 0.69 (0.29, 1.67); P = 0.415 | 0.31 (0.11, 0.82); P = 0.018 | **2.19 (1.23, 3.92); P = 0.008** |  |
| Quintiles | Q1 | Q2 | Q3 | Q4 | **Q5** |
| *N* (incident IPH) | 499(87) | 48(3) | 63(6) | 53(7) | **73(21)** |
| Odds ratio (95% CI) | Reference | 0.37 (0.1, 1.36); P = 0.134 | 0.51 (0.21, 1.26); P = 0.146 | 0.77 (0.3, 1.98); P = 0.582 | **2.5 (1.35, 4.65); P = 0.004** |

IPH = intraplaque haemorrhage, METh-week = metabolic equivalent task hours per week.

The literature-based cutoff values of high volume for total and moderate to vigorous physical activity were based on the Physical Activity Guidelines Advisory Committee Report, as adapted by previous studies (Laura et al., JAMA Cardiology, 2019; Kerem et al., JAMA Cardiology, 2024).

Sex-specific tertiles, quartiles, and quintiles are applied for total, moderate to vigorous, and moderate physical activity. For vigorous physical activity, because most participants did not engage in any vigorous physical activity, the lowest group was defined as individuals who reported none, and sex-specific median split, tertiles, and quartiles were created among those who did engage in vigorous physical activity.

Odds ratios were obtained using generalised estimated equation with a binomial distribution and a logit link function, adjusting for the follow-up time between two MRI measurements, sex, baseline age, body mass index, smoking status, Rotterdam Study sub-cohort, educational level, hypercholesterolemia, hypertension, diabetes, and maximum intima-media thickness.

**Supplemental Table 11.** The association between physical activity and incident lipid-rich necrotic core after excluding employed participants (n = 564)

| Total physical activity | | | | | |
| --- | --- | --- | --- | --- | --- |
| Literature-based cutoff | < 25 MET-h/week | >= 25 & < 50 MET-h/week | >= 50 MET-h/week |  |  |
| *N* (incident LRNC) | 205(83) | 95(38) | 243(112) |  |  |
| Odds ratio (95% CI) | Reference | 0.9 (0.54, 1.52); P = 0.700 | 1.21 (0.81, 1.81); P = 0.350 |  |  |
| Tertiles | T1 | T2 | T3 |  |  |
| *N* (incident LRNC) | 199(79) | 161(66) | 183(88) |  |  |
| Odds ratio (95% CI) | Reference | 1.03 (0.66, 1.6); P = 0.903 | 1.33 (0.86, 2.05); P = 0.203 |  |  |
| Quartiles | Q1 | Q2 | Q3 | Q4 |  |
| *N* (incident LRNC) | 145(52) | 130(60) | 142(56) | 126(65) |  |
| Odds ratio (95% CI) | Reference | 1.41 (0.84, 2.36); P = 0.196 | 1.08 (0.65, 1.79); P = 0.756 | 1.76 (1.05, 2.93); P = 0.032 |  |
| Quintiles | Q1 | Q2 | Q3 | Q4 | **Q5** |
| *N* (incident LRNC) | 118(45) | 112(49) | 95(37) | 132(55) | **86(47)** |
| Odds ratio (95% CI) | Reference | 1.2 (0.68, 2.1); P = 0.528 | 0.95 (0.53, 1.71); P = 0.864 | 1.06 (0.62, 1.83); P = 0.823 | **1.9 (1.05, 3.44); P = 0.035** |
| Moderate to vigorous physical activity | | | | | |
| Literature-based cutoff | < 25 MET-h/week | >= 25 & < 50 MET-h/week | **>= 50 MET-h/week** |  |  |
| *N* (incident LRNC) | 257(96) | 127(56) | **159(81)** |  |  |
| Odds ratio (95% CI) | Reference | 1.25 (0.79, 1.98); P = 0.350 | **1.69 (1.09, 2.64); P = 0.020** |  |  |
| Tertiles | T1 | T2 | T3 |  |  |
| *N* (incident LRNC) | 198(74) | 171(73) | 174(86) |  |  |
| Odds ratio (95% CI) | Reference | 1.17 (0.75, 1.83); P = 0.489 | 1.52 (0.97, 2.37); P = 0.069 |  |  |
| Quartiles | Q1 | Q2 | Q3 | **Q4** |  |
| *N* (incident LRNC) | 143(56) | 142(52) | 127(56) | **131(69)** |  |
| Odds ratio (95% CI) | Reference | 0.91 (0.54, 1.51); P = 0.707 | 1.22 (0.73, 2.04); P = 0.443 | **1.71 (1.02, 2.88); P = 0.041** |  |
| Quintiles | Q1 | Q2 | Q3 | Q4 | Q5 |
| *N* (incident LRNC) | 119(50) | 115(37) | 104(48) | 114(52) | 91(46) |
| Odds ratio (95% CI) | Reference | 0.68 (0.38, 1.2); P = 0.182 | 1.16 (0.66, 2.03); P = 0.602 | 1.04 (0.6, 1.8); P = 0.884 | 1.48 (0.83, 2.65); P = 0.186 |
| Moderate physical activity | | | | | |
| Tertiles | T1 | T2 | T3 |  |  |
| *N* (incident LRNC) | 190(71) | 180(84) | 173(78) |  |  |
| Odds ratio (95% CI) | Reference | 1.38 (0.89, 2.14); P = 0.151 | 1.28 (0.81, 2.02); P = 0.287 |  |  |
| Quartiles | Q1 | Q2 | Q3 | Q4 |  |
| *N* (incident LRNC) | 147(60) | 141(52) | 126(63) | 129(58) |  |
| Odds ratio (95% CI) | Reference | 0.86 (0.52, 1.43); P = 0.568 | 1.29 (0.77, 2.15); P = 0.328 | 1.12 (0.67, 1.88); P = 0.669 |  |
| Quintiles | Q1 | Q2 | Q3 | Q4 | Q5 |
| *N* (incident LRNC) | 119(50) | 114(37) | 103(52) | 113(51) | 94(43) |
| Odds ratio (95% CI) | Reference | 0.67 (0.38, 1.18); P = 0.163 | 1.36 (0.77, 2.4); P = 0.294 | 1.02 (0.59, 1.79); P = 0.935 | 1.07 (0.59, 1.92); P = 0.833 |
| Vigorous physical activity | | | | | |
| Tertiles | T1 | T2 | **T3** |  |  |
| *N* (incident LRNC) | 386(155) | 81(35) | **76(43)** |  |  |
| Odds ratio (95% CI) | Reference | 0.96 (0.58, 1.58); P = 0.871 | **2.04 (1.17, 3.54); P = 0.012** |  |  |
| Quartiles | Q1 | Q2 | Q3 | **Q4** |  |
| *N* (incident LRNC) | 386(155) | 40(20) | 60(24) | **57(34)** |  |
| Odds ratio (95% CI) | Reference | 1.34 (0.69, 2.58); P = 0.384 | 0.87 (0.47, 1.61); P = 0.662 | **2.24 (1.2, 4.18); P = 0.011** |  |
| Quintiles | Q1 | Q2 | Q3 | Q4 | **Q5** |
| *N* (incident LRNC) | 386(155) | 29(11) | 52(24) | 35(17) | **41(26)** |
| Odds ratio (95% CI) | Reference | 0.94 (0.42, 2.08); P = 0.879 | 0.98 (0.53, 1.81); P = 0.941 | 1.45 (0.68, 3.07); P = 0.334 | **2.84 (1.37, 5.9); P = 0.005** |

LRNC = lipid-rich necrotic core, METh-week = metabolic equivalent task hours per week.

The literature-based cutoff values of high volume for total and moderate to vigorous physical activity were based on the Physical Activity Guidelines Advisory Committee Report, as adapted by previous studies (Laura et al., JAMA Cardiology, 2019; Kerem et al., JAMA Cardiology, 2024).

Sex-specific tertiles, quartiles, and quintiles are applied for total, moderate to vigorous, and moderate physical activity. For vigorous physical activity, because most participants did not engage in any vigorous physical activity, the lowest group was defined as individuals who reported none, and sex-specific median split, tertiles, and quartiles were created among those who did engage in vigorous physical activity.

Odds ratios were obtained using generalised estimated equation with a binomial distribution and a logit link function, adjusting for the follow-up time between two MRI measurements, sex, baseline age, body mass index, smoking status, Rotterdam Study sub-cohort, educational level, hypercholesterolemia, hypertension, diabetes, and maximum intima-media thickness.

**Supplemental Table 12.** Hazards for first-ever stroke by the presence of vulnerable carotid plaque components after excluding employed participants (n = 1148)

|  | Full cohort | |  | | Subgroup without vulnerable components | Subgroup with vulnerable components |
| --- | --- | --- | --- | --- | --- | --- |
| per 20 Met-h/week | *Hazards ratio* (95% CI) | *P* interaction |  | *Hazards ratio* (95% CI) | | *Hazards ratio* (95% CI) |
| Total physical activity | 0.95 (0.87, 1.04) | **0.017** |  | 0.70 (0.54, 0.91) | | 1.00 (0.91, 1.11) |
| Moderate to vigorous physical activity | 0.95 (0.85, 1.07) | 0.127 |  | 0.73 (0.54, 0.99) | | 1.00 (0.89, 1.12) |
| Moderate physical activity | 0.96 (0.85, 1.10) | 0.270 |  | 0.77 (0.55, 1.07) | | 1.00 (0.88, 1.14) |
| Vigorous physical activity | 0.88 (0.65, 1.21) | 0.142 |  | 0.32 (0.08, 1.33) | | 1.02 (0.74, 1.41) |

CI = confidence interval, MET = metabolic equivalent task.

Hazards ratios were estimated using cause-specific Cox models, adjusting for sex, baseline age, body mass index, smoking status, RS-cohort index, education, hypercholesterolemia, hypertension, diabetes, maximum intima-media thickness. P for interaction was obtained by including an interaction term of physical activity and IPH in the model. The median of follow-up time is 9.5 years (inter-quartile range: 8.2 years, 11.8 years).

The presence of vulnerable components was defined as the presence of intraplaque haemorrhage or/and lipid-rich necrotic core.
